# Impact of newborn screening on the reported incidence and clinical outcomes associated with medium- and long-chain fatty acid oxidation disorders

**DOI:** 10.1038/s41436-020-01070-0

**Published:** 2021-01-25

**Authors:** Deborah Marsden, Camille L. Bedrosian, Jerry Vockley

**Affiliations:** 1 Ultragenyx Pharmaceutical Inc, Novato, CA USA; 2 Department of Pediatrics, University of Pittsburgh School of Medicine, Pittsburgh, PA USA

## Abstract

Fatty acid oxidation disorders (FAODs) are potentially fatal inherited disorders for which management focuses on early disease detection and dietary intervention to reduce the impact of metabolic crises and associated spectrum of clinical symptoms. They can be divided functionally into long-chain (LC-FAODs) and medium-chain disorders (almost exclusively deficiency of medium-chain acyl–coenzyme A dehydrogenase). Newborn screening (NBS) allows prompt identification and management. FAOD detection rates have increased following the addition of FAODs to NBS programs in the United States and many developed countries. NBS-identified neonates with FAODs may remain asymptomatic with dietary management. Evidence from numerous studies suggests that NBS-identified patients have improved outcomes compared with clinically diagnosed patients, including reduced rates of symptomatic manifestations, neurodevelopmental impairment, and death. The limitations of NBS include the potential for false-negative and false-positive results, and the need for confirmatory testing. Although NBS alone does not predict the consequences of disease, outcomes, or management needs, subsequent genetic analyses may have predictive value. Genotyping can provide valuable information on the nature and frequency of pathogenic variants involved with FAODs and their association with specific phenotypes. Long-term follow-up to fully understand the clinical spectrum of NBS-identified patients and the effect of different management strategies is needed.

## INTRODUCTION

Fatty acid oxidation disorders (FAODs) are inherited disorders that manifest with clinical presentations (most notably hypoketotic hypoglycemia, cardiomyopathy, and myopathy with recurrent rhabdomyolysis) related to commonly affected organs (i.e., liver, heart, and skeletal muscle).^1^ Long-chain FAODs (LC-FAODs) are caused by defects in the mitochondria carnitine shuttle or enzymes involved in the β-oxidation of long-chain fatty acids.^1^ Medium-chain acyl–coenzyme A (CoA) dehydrogenase (MCAD) deficiency is the most common inherited FAOD, especially in Caucasian populations of Northern European descent.^1^ Short-chain acyl-CoA dehydrogenase (SCAD) deficiency is often caused by compound heterozygosity or homozygosity for one of two common polymorphisms. While these variants may result in reduced enzyme activity, SCAD deficiency is generally considered a benign condition.^1^ MCAD deficiency and LC-FAODs are potentially fatal, and management focuses on prevention of metabolic crises by avoiding fasting. For patients with LC-FAODs, supplementation with medium-chain triglycerides can reduce the impact of clinical symptoms.

Newborn screening (NBS) allows prompt identification and management of disorders with early onset and serious, life-threatening consequences, including many inborn errors of metabolism. Early NBS programs tested for a relatively small number of conditions, typically initially limited to phenylketonuria. NBS has since expanded to include a wide range of conditions, with test menus specific to countries and regions. In the United States, the Recommended Uniform Screening Panel developed and curated by an advisory committee to the Secretary of the Department of Health and Human Services^2^ has helped standardize NBS. The addition of FAODs to NBS programs began in the mid-1990s as they became recognized as a cause of sudden infant death and other life-threatening consequences in the newborn period.^3,4^ In patients with LC-FAOD, one of the main suspected causes of sudden infant death is cardiac ventricular arrhythmia.^5^ However, this cause of death is mainly a diagnosis by exclusion as it is only detectable by electrocardiogram, and unless the patient is already hospitalized, cannot be confirmed. It has since been suggested that FAODs should be suspected in all cases of sudden infant death.^4,6,7^ Screening is dependent on detecting abnormal concentrations of metabolites in the acylcarnitine profile in blood through the use of tandem mass spectrometry. NBS for FAODs is available in the United States and many developed countries, although geographical differences exist in implementation (see Table S1). In 2008, an expert symposium held in Germany examined NBS programs that include FAODs from various perspectives, including availability in different countries/regions, epidemiological data, test performance, and follow-up practices.^3^ Here, outcomes research findings suggested notable reductions in metabolic decompensations and death associated with identification of MCAD deficiency via NBS. Further evidence suggested that the cost-effectiveness of NBS for FAODs compared favorably with other preventive programs.^3^ Since then, additional evidence in a variety of settings has accumulated related to the epidemiology and impact of NBS for FAODs. In the present review, we examine available evidence related to the epidemiology of FAODs, with a focus on LC-FAODs, as well as the impact of NBS on clinical outcomes, including mortality.

## EPIDEMIOLOGY OF FAODs BY COUNTRY AND REGION

The incidence or prevalence of all FAODs and specific subtypes has been reported from numerous pilot and nationwide NBS programs (Table 1). Considerable heterogeneity exists in the size and characteristics of the populations examined, details of screening test methods, cutoffs for recall, and use of additional confirmatory tests. Consequently, incidence data should be considered indicative in the context of the unique features of individual reports. For example, reported incidence may be affected by minor differences in absolute numbers of detected cases in small carrier screening populations. As a result, detailed comparisons of incidence data between countries and regions are difficult to make. However, some generalizations are possible.

**Table 1:** Incidence of FAODs overall, including specific LC-FAOD (VLCAD deficiency and LCHAD/TFP deficiency).

				Incidence per 100,000 births			
Country/region	Year(s)	Study/registry details	Patients screened	All FAODs (LC-FAOD, MCAD, SCAD)	VLCAD deficiency	LCHAD/TFP deficiency	Comments and additional findings
**Asia/Oceania**							
Australia, Victoria^65^	2002–2005	Case series report of NBS	189,000		3.2^a^		Variant analysis is important to avoid false-negative diagnoses in asymptomatic newborns
Australia, New South Wales^22^	1998–2010 vs. 2010–2015	Comparison of clinical diagnosis/NBS vs. NBS with region 4 tool	2,100,000; 528,000		0.7 (clinical diagnosis/NBS) vs. 2.1 (NBS with region 4 tool)		Region 4 scores can distinguish a clinically affected VLCAD deficiency population from an NBS-identified cohort at extremely low risk of having symptoms
Australia, New South Wales, and the Australian Capital Territory^11^	1974–2002	Comparison of detection rates of 31 IEM before and after NBS (1998)	362,000	0.9–3.2 (1974–1998) 8.0 (1998–2002)			FAOD detection rate significantly increased after NBS compared with clinical diagnosis (*p* = 0.007)
China^12^	2014–2015	Report of NBS conducted in 100,077 neonates from Jining	100,077	11.0			Report recommended a nationwide NBS program, especially in poor areas of China
China^14^	2009–2016	Report of NBS program from the Zhejiang province	1,861,262	6.5^a^	0.16		Most patients identified with FAODs, including LCHAD, MCAD, and SCAD, were asymptomatic with normal growth and development after early intervention and management
India^23^	Not reported	Analysis of 903S card for IEM among 200,000 newborns in New Delhi	200,000		0.5		Higher incidence of conditions overall ascribed to high degree of consanguinity and epigenetic factors
Japan^10^	1997–2015	Comparison of IEM incidence in Asian countries and Germany via selective screening and NBS	3,360,000	3.3	1.1	0.12	Identification of FAOD by selective screening and expanded NBS were distinct
Singapore^9^	2006–2014	Report of pilot and full national NBS program	177,267	15.2	2.3		Use of absolute cutoffs vs. initial use of 99th percentile significantly reduced high pilot phase recall rate
South Korea^13^	2001–2003	NBS experience including 37,817 neonates	37,817	10.6	2.6		The relatively normal development noted among identified children (except for deceased) demonstrates the effectiveness of NBS
South Korea^10^	2000–2015	Comparison of IEM incidence in Asian countries and Germany via selective screening and NBS	3,440,000	0.9	0.3	0.09	
Taiwan^8^	2003–2012	Report of NBS program among >800,000 Chinese neonates screened at the National Taiwan University Hospital		2.9	0.25		Report highlighted false-negative cases and the dangers of rescreening because repeat tests can have normal results
Taiwan^10^	2001–2014	Comparison of IEM incidence in Asian countries and Germany via selective screening and NBS	1,390,000	2.9	0.07		
New Zealand^34^	2004–2009	Retrospective analysis comparing rates of IEM detection before (January 2004–December 2006) and after (December 2006–December 2009) expanded NBS	Pre-NBS: 175,000 births; most diagnosed symptomatically Post-NBS: 185,000 births		0.57 (pre-NBS) vs. 0.54 (post-NBS)		NBS led to a dramatic increase in detection of IEM cases overall (1 per 12,000 to 1 per 4400 for all conditions), which is not reflected in FAODs due to low numbers
**Europe**							
Austria^16^	2002–2009	Report of Austrian NBS of 622,489 newborns screened for >20 diseases	622,489	7.6	1.1		Report highlights high incidence of FAODs overall, mostly MCAD deficiency, and the need for confirmatory testing to minimize the risk of false-positive cases
Czech Republic^28^	2002–2010	Report of results of nationwide NBS in newborns screened for various IEM, including FAODs	106,522		0.7	0.68^a^	Cumulative detection rate increased with expansion of the test menu
Czech Republic^35^	Pre-2009 and 5 years post-2009	Comparison of NBS before and after expansion to include MCAD deficiency and LCHAD deficiency	661,000			0.7 (pre-NBS) vs. 1.5 (post-NBS)	Expanding NBS to include MCAD deficiency and LCHAD deficiency significantly increased detection rate and improved clinical outcomes
Czech Republic^24^	2002–2016	Retrospective analysis of NBS in normal and low–birth weight neonates	777,100–1,277,283		0.25^a^	1.3^a^	Association noted between low birth weight and LCHAD deficiency, although cause and effect were unclear
Estonia^66^	2004–2007	Cohort–control study of 1040 newborn blood spot samples screened for the c.1528G>C variant	1,040			1.1^a^	2 of 425 patients tested using acylcarnitine had an abnormal profile typical of LCHAD deficiency
Germany^67^		Follow-up of 8 infants identified by NBS using enzyme and/or mutational analysis	8		0.8		Incidence is estimate for Germany based on personal communication
Germany^17^	1999–2000	Active surveillance of symptomatic infants over 2 years	844,575	3.2	0.2	0.36	Most cases presented in the first year of life by acute metabolic decompensation
Germany^10^	2002–2015	Comparison of IEM incidence in Asian countries and Germany via selective screening and NBS	7,510,000	11.1	1.3	0.57	
The Netherlands^25^	1963–2010	Retrospective comparison of identification pre and post-NBS (from 2007)	560,000		0.3 (pre-NBS) vs. 1.3 (post-NBS)		c.848TNC (p.V283A) was the most frequently encountered variant
Norway^19^	2012–2018	Report of 6 years of experience with expanded NBS	357,436	8.7			Positive predictive value of testing improved over the 6 years examined
Poland^68^	1992–2009	Analysis of diagnostic approach mode on detection rate and mortality risk in LCHAD deficiency	658,492			0.87 (molecularly confirmed symptomatic cases) 0.91 (NBS)	Mortality rate greatly improved by NBS vs. selective screening or differential diagnosis
Poland^32^	2008	Analysis of 6,854 neonatal blood samples from different regions of Poland screened for the c.1528G>C variant in the *HADHA* gene	6,854			5.9 (Pomeranian) 0.84 (Poland)	Geographically skewed distribution of the c.1528C allele in the northern Pomeranian province of Poland justifies screening for LCHAD deficiency in neonates born in northern Poland
Portugal^20^	2004–2012	Report of expanded NBS program in Portugal	737,902	16.4			
Portugal and Spain^18^	Not reported	Report of six NBS programs in Portugal and Spain (46.2% of all annual births in region)	1,672,286	12.6^a^	0.47^a^	0.72^a^	High prevalence of MCAD deficiency with high homogeneity for the c.985A>G variant among the Gypsy population
Slovenia^27^	2013–2014	Pilot study of expanded NBS, including NGS in 10,048 neonates compared with clinically detected cases (1999–2013)	10,048		10.0 (NBS) <0.34 (clinical diagnosis)	0 (NBS) 0.34 (clinical diagnosis)	NGS enabled the differentiation between affected patients and heterozygotes while improving the turnaround time of genetic analysis
**United States**							
California, Oregon, Washington, and Hawaii^26^	2005–2009	Retrospective analysis of newborns with elevated C14:1-acylcarnitine on NBS with available confirmatory testing and clinical information	2,802,504		1.9		Demonstrates necessity of comprehensive and consistent long-term NBS follow-up systems
Minnesota^3^	2004–2008	Expert panel report of NBS programs, including 378,272 neonates screened in Minnesota	378,272		1.1	0.13	Report noted the importance of minimizing the false-positive rate with expanded programs
North Carolina^15^	1997–2005	Report of NBS program (tandem mass spectrometry) incorporating confirmatory testing	944,078	10.5	1.3	0.33	Success of the NBS program was dependent on comprehensive follow-up protocol
**Middle East**							
Saudi Arabia^21^	2001–2014	Retrospective cohort study of 110,601 live births examined for IEM by clinical suspicion or NBS from 2011	110,601	4.0			Report noted the high incidence of IEM overall in this population, the phenotype of VLCAD deficiency cases, and the value of confirmatory testing
Qatar^3^	2004–2008	From an expert panel report of NBS programs, including 71,069 neonates screened in Qatar	71,069		1.4		
Saudi Arabia^29^	2013–2017	Retrospective study of the Saudi national NBS program	199,143		31.1		High incidence of VLCAD deficiency was largely restricted to one tribe in Jawf province and related to consanguineous marriage
**Multiple**							
Australia, Germany, and the United States^3^	2004–2008	From an expert panel report of NBS programs	5,256,999	10.8	1.2	0.40 (LCHAD) 0.13 (TFP)	

*IEM* inborn error of metabolism, *LC-FAOD* long-chain fatty acid oxidation disorder, *LCHAD* long-chain 3-hydroxyacyl–coenzyme A dehydrogenase, *MCAD* medium-chain acyl–coenzyme A (CoA) dehydrogenase, *NBS* newborn screening, *NGS* next-generation sequencing, *SCAD* short-chain acyl-CoA dehydrogenase, *TFP* trifunctional protein, *VLCAD* very long–chain acyl–coenzyme A dehydrogenase.

^a^Supporting references available upon request.

Epidemiologic studies of NBS populations indicate that the combined incidence of all FAODs ranges from 0.9 to 15.2 per 100,000. Very long–chain acyl-CoA dehydrogenase (VLCAD) deficiency is the most prevalent LC-FAOD in most populations, with incidences ranging from 0.07 to 1.9 per 100,000, whereas other LC-FAODs have a low incidence (<1.0 per 100,000).^8–14^ Occasional outlier figures have been published that are not explicable based on population characteristics, test methods, sample size, or other factors.

The incidence of all FAODs among Asian populations is generally lower than in non-Asian populations, ranging from 0.9 to 4.9 per 100,000 in large carrier screening populations in China, Japan, South Korea, and Taiwan.^8,10,14^ In contrast, a relatively small carrier screening population (*N* = 37,817 neonates) over 2 years in a single laboratory in Seoul identified an FAOD incidence of 10.6 per 100,000.^13^ However, only four patients were identified, demonstrating the effect a small cluster of cases may have on the reported incidence in a small population.^13^ Another study in Singapore reported an FAOD incidence of 15.2 per 100,000, which is higher than other studies in Asian populations.^9^ These results may have been skewed during the pilot phase program, which used the 99th percentile of the analyte distribution of the newborn population as a cutoff for recall. Subsequent use of absolute cutoff values reduced this high recall rate. A study in rural China also reported an unusually high FAOD incidence (11.0 per 100,000) for Asian countries.^12^ In Taiwan, a study of NBS determined the incidence to be 4.53 cases of FAODs per 100,000 live births.^8^ In an Australian study, the incidence of FAODs before the introduction of NBS (0.9–3.2 per 100,000) more than doubled to 8.0 per 100,000 following introduction of NBS in 1998, consistent with a subsequent combined report from Australia, Germany, and the United States (10.8 per 100,000).^3,11^

The incidence of FAODs in several European and US studies ranged from 6.0 to 16.4 per 100,000.^10,15–19^ The highest incidences in Europe were reported in Spain and Portugal, which have a high incidence of MCAD deficiency among mainly Gypsy populations, likely secondary to a founder effect and consanguinity.^18,20^ The incidence of FAODs (4.0 per 100,000) reported in a single-center retrospective review in Saudi Arabia over a 13-year period was similar to that of Asian populations.^21^

Among the LC-FAODs, VLCAD deficiency is the most common. It is an autosomal recessive disorder caused by *ACADVL* variants, leading to impairment of the first intramitochondrial step of the catabolism of long-chain fatty acids.^1^ The incidence of VLCAD deficiency based on NBS reports varies among countries and regions, though much less than MCAD deficiency (Table 1). Incidence of VLCAD deficiency in Asia and Australia is similar, though use of the region 4 Stork Collaborative Project postanalytical NBS tool in the latter led to a threefold increase in the previous detection rate based on clinical diagnosis or prior NBS algorithms, underscoring the tool’s ability to distinguish a clinically affected VLCAD-deficient population from an NBS-identified cohort at extremely low symptomatic risk.^9,10,12–14,22,23^ Studies of European, US, and Qatar populations have identified incidences similar to Asia and Australia.^3,10,15–18,24–28^ However, the Eastern and Jawf provinces of Saudi Arabia reported a much higher incidence (31.1 per 100,000), which is attributed to a high rate of consanguineous marriage and a founder effect.^29^ In one study, patients identified by NBS generally had higher levels of enzyme activity and a higher overall long-chain fatty acid oxidation flux in cultured fibroblasts than those reported before NBS implementation.^30^ These findings suggest that NBS identifies patients with milder genetic and biochemical phenotypes, who may have improved long-term outcomes with appropriate management. A retrospective analysis from the Netherlands questioned whether the rise in asymptomatic VLCAD deficiency was attributable to underdiagnosis of an otherwise relatively common condition or if patients detected by NBS might never become symptomatic unless experiencing significant catabolic stress.^25^

Long-chain 3-hydroxyacyl-CoA dehydrogenase (LCHAD) and trifunctional protein (TFP) deficiencies can present with serious, rapidly progressive cardiomyopathy, hypoketotic hypoglycemia and liver dysfunction, and rhabdomyolysis, among other manifestations.^1^ Almost all cases of isolated LCHAD deficiency are caused by a c.1528G>C variant of the *HADHA* gene encoding the α subunit of TFP. The incidence of LCHAD deficiency in most studies was <1.5 per 100,000, with higher incidences noted in European compared with Asian countries or the United States (Table 1). This is consistent with a previous meta-analysis of 16 studies of LCHAD deficiency representing a carrier screening population of 13 million, which reported a prevalence of 0.41–0.91 per 100,000.^31^ One study that examined 6,854 neonatal blood samples from different regions of Poland revealed a geographically skewed distribution of the c.1528G>C allele in the northern Pomeranian province (incidence 5.9 per 100,000).^32^ Deficiency of all three TFP enzymes (LCHAD, long-chain enoyl-CoA hydratase, and 3-ketoacyl-CoA thiolase) is caused by almost all other variants of either the *HADHA* or *HADHB* genes. TFP deficiency is considered extremely rare. Our review supported this finding with an incidence of 0.13 per 100,000 reported in a single study in multiple countries.^3^

Deficiencies of the carnitine cycle include defects in carnitine palmitoyltransferase-1 (CPT-1), carnitine–acylcarnitine translocase (CACT), and carnitine palmitoyltransferase-2 (CPT-2). These autosomal recessive disorders affect mitochondrial import of long-chain fatty acyl-CoAs. Studies in this review confirmed the rare nature of these disorders, with reported incidences <0.33 per 100,000 in most studies (Table 2). A Hong Kong study reported a high incidence of CACT deficiency (3.3 per 100,000), but this involved a single confirmed case in a relatively small carrier screening population (30,448 neonates).^33^ Similarly, three unrelated cases of CACT deficiency from different ethnic groups were reported in a New Zealand NBS program of 185,000 neonates screened from 2006 to 2009.^34^ This unlikely cluster led to an incidence of 1.6 per 100,000 and should be viewed as an outlier against the background of most other reported incidences.^3^

**Table 2:** Incidence of LC-FAODs (CPT-1, CPT-2, and CACT).

Country/registry	Year	Incidence per 100,000 births		
		CPT-1	CPT-2	CACT
**Asia/Oceania**				
Australia^11^	1998–2002			0.28
China^14^	2009–2016	0.11^a^	0.11^a^	
Hong Kong^33^	2013–2016			3.3
India^23^		1.0		
Japan^10^		0.24	0.39	
Singapore^9^	2006–2014			0.56
Taiwan^8^	2003–2012	0.13	0.25	
Taiwan^10^		0.14	0.14	
New Zealand^34^	2004–2006 2006–2009			0 (2004–2006) 1.62 (2006–2009)
**Europe**				
Germany^17^	1999–2000		0.12	
Germany^10^		0.10	0.03	0.01
Norway^19^	2012–2018		0.28	
Portugal and Spain^18^		0.17	0.35	
**United States**				
Minnesota^3^	2004–2008		0.26	
North Carolina^15^	1997–2005		0.11	
**Multiple**				
Australia, Germany, and the United States^3^	2004–2008	0.05–0.13	0.05–0.13	0.05–0.13

*CACT* carnitine–acylcarnitine translocase, *CPT-1* carnitine palmitoyltransferase-1, *CPT-2* carnitine palmitoyltransferase-2, *LC-FAOD* long-chain fatty acid oxidation disorder.

^a^Supporting references available upon request.

NBS has led to a number of important recognitions regarding FAODs. First, FAOD detection rates have increased following the widespread introduction of FAODs to NBS. In an Australian study of NBS data for inborn errors of metabolism, overall rates of FAOD detection increased significantly (*p* = 0.007) from 0.9–3.2 to 8.0 per 100,000 births with the introduction of NBS.^11^ In the Czech Republic, a study demonstrated a similar significant increase in the identification of patients with LCHAD from 0.7 to 1.5 per 100,000 births following the utilization of NBS.^35^ In a Dutch study spanning from 1963 through 2010, the incidence of VLCAD deficiency increased from 0.3 to 1.3 per 100,000 births after NBS was introduced.^25^ While the use of NBS clearly increases the detection of patients with FAODs allowing for early disease management, it is important to note that disease detection alone cannot predict whether these patients will go on to develop symptoms or remain asymptomatic for life without intervention.

Additionally, the use of NBS in detecting FAODs highlights the limitations of NBS, including the potential for both false-negative and false-positive results, the need for confirmatory testing, and the effect of the chosen cutoff values for acylcarnitine levels and recall for retesting. The possibility of false-negative (“missed”) cases is concerning, especially in late-screened babies in whom analyte levels may have normalized. While limited data exist surrounding such false-negative tests, several studies have reported cases. In a study of 39 Chinese patients, 5 were identified as having FAODs after negative NBS results.^8^ Here, cases were predominantly identified by genetic analysis following metabolic decompensation or familial diagnosis. Another study describes a patient who was missed by initial NBS for CPT-2 deficiency.^36^ Researchers sought to establish an alternative diagnostic criterion focused on the ratio of C16 + C18:1/C2 to enhance the diagnostic accuracy patients with CPT-2 deficiency. In a study of the use of NBS in inborn errors of metabolism, rates of detection of 31 disorders including FAODs were examined.^11^ Of 57 patients with inborn errors of metabolism identified following the introduction of NBS, 15 were diagnosed clinically, and 7 of these were negative on NBS. One of the false-negative patients later presented with hypoglycemia due to VLCAD deficiency.^11^ Lastly, in a study of 14 Austrian patients with LCHAD deficiency, two patients were reported who had negative NBS results and subsequently presented with acute metabolic decompensations.^37^ From the limited availability of false-negative NBS outcomes in patients with FAODs, it is clear that these instances are rare. However, their existence indicates that there is a subset of patients with FAODs who are missed by NBS and experience delays in the initiation of disease management. While it is true that some of these patients may never go on to develop symptoms, others may experience potentially life-threatening metabolic decompensations, and it is therefore important to increase diagnostic efficiency to identify them. In addition, false-positive NBS outcomes may skew the overall incidence and prevalence rates of FAODs. Further standardization of NBS amplifies this potential problem, as a rate of misdiagnosis is applied universally to a global population, resulting in a potentially exaggerated incidence rate. The differentiation of heterozygotes (unaffected heterozygotes) from infants with potentially mild or late-onset disease can be challenging, and difficulties in obtaining confirmatory testing (either molecular or enzymatic) often accentuate this problem.^8,36,37^

## IMPACT OF NBS ON CLINICAL OUTCOMES

Despite limitations, NBS has led to improved clinical outcomes for infants identified with FAODs, allowing neonates definitively identified with an FAOD to promptly receive appropriate diet modification and other management tailored specifically to their individual FAOD, as well as counseling of caregivers. As summarized below, numerous studies show that neonates with NBS-identified FAODs may remain asymptomatic with appropriate management and may have better outcomes compared with individuals identified via clinical symptoms or selective screening. The improvement is most striking with MCAD deficiency, where mortality is nearly eliminated following identification by NBS. While patients with LC-FAODs are less likely to develop symptoms as newborns and may remain asymptomatic as infants, manifestations typically emerge later in life, so ongoing monitoring and management are warranted.^38^

Numerous studies have examined the clinical course of patients with FAODs identified by NBS.^38–41^ In the earliest of these, eight asymptomatic patients from Germany and the United States with possible VLCAD deficiency identified by NBS (*n* = 7; 2–3 days at analysis) or family screening (*n* = 1; 3 years at analysis) underwent enzyme and/or genetic analysis to confirm and characterize their diagnosis.^38^ Molecular analysis identified missense variants in seven of eight patients, and biochemical assays found that VLCAD activity was reduced to 6–11% of normal in four of eight patients. Clinical signs (e.g., hepatopathy, cardiomyopathy, arrhythmias, or muscular hypotonia) were not present in the cohort throughout follow-up (range 2 months to 3.5 years).^38^

A large retrospective study used the US Inborn Errors of Metabolism Information System data collection tool to analyze outcomes among 52 infants positively identified with VLCAD deficiency via NBS.^41^ Age at study entry ranged from 11 days to 11.4 years, and the study included 378 person-years of follow-up. Initial C14:1 acylcarnitine levels were higher in symptomatic (*M* = 3.70 μM) compared with asymptomatic patients (*M* = 1.94 μM; *p* < 0.002), suggesting a correlation with disease presentation. Elevated creatinine kinase (≥250 IU/L) was common, and rhabdomyolysis was present in 11 of 14 infants with elevated creatinine kinase levels. Cardiomyopathy was uncommon. Neonatal complications were reported in 9 (17%) of 52 patients, and thus most NBS-identified newborns remained asymptomatic. The authors discussed the use of combinations of confirmatory tests, including molecular analysis, fibroblast acylcarnitine profiling, and white blood cell or fibroblast enzyme assay. Functional studies were considered important in patients with uncharacterized variants.^41^

The challenge of determining outcomes among asymptomatic infants identified early in the disease course, many of whom remain asymptomatic during initial follow-up, is highlighted in a retrospective review describing the long-term experience of 23 patients with VLCAD deficiency in Australia. Babies identified since the addition of FAODs to NBS programs were followed for a median of 104 months (range 14 months to 16.6 years).^39^ The original management protocol consisted of a very low natural fat diet (~10% of intake) and supplementation with medium-chain triglycerides, but this was relaxed to a natural diet containing ~30% of intake as long-chain fats after age 5 years for asymptomatic patients. Metabolic stability, growth, development, and cardiac function were satisfactory in all patients, with no episodes of encephalopathy or hypoglycemia. Only 14 of 65 hospital admissions after diagnosis were due to metabolic decompensation, and three patients had episodes of muscle pain with or without rhabdomyolysis. This study noted the difficulty in predicting disease presentation, outcomes, and management needs from initial NBS results, but acknowledged that genetic analysis may have predictive value.^39^

A retrospective analysis covering 4 years of genetic screening at three centers in Spain examined parameters that distinguished unambiguous VLCAD deficiency cases from heterozygotes.^40^ Among 36 cases identified as positive by NBS, 12 cases were confirmed based on subsequent increases in C14:1 acylcarnitine and its ratio to other metabolites, the presence of two pathogenic variants, and enzyme activity in lymphocytes <12% of the intra-assay control. Eleven patients remained asymptomatic with appropriate nutritional therapy after a mean follow-up of 30 months, and one was symptomatic.^40^ These results highlight the effectiveness of NBS in detecting disease and positively impacting outcomes while emphasizing the role of additional biochemical, enzymatic, and comprehensive gene testing in distinguishing heterozygotes and false positives.

As a cautionary note, a long-term retrospective analysis of 83 clinical charts from the Portuguese national NBS program highlights the possibility of acute manifestations, including death, despite NBS implementation.^42^ Among the 83 records, 19 patients had acute decompensations, including six patients who developed symptoms 2–7 days after birth and before NBS was performed or results were available.^42^ Such episodes occurred in patients with MCAD deficiency, but were more frequent in those with LC-FAODs, who showed a wide range of clinical manifestations. These observations support the need for early screening and prompt disease management to prevent potentially life-threatening metabolic decompensation events, which could occur within the first few days of life. Indeed, recommendations have been made that time-critical conditions be reported to the patient’s physician within 5 days to minimize this risk.^43–45^

### Outcomes for clinically diagnosed vs. NBS-identified patients

**Fig. 1:**
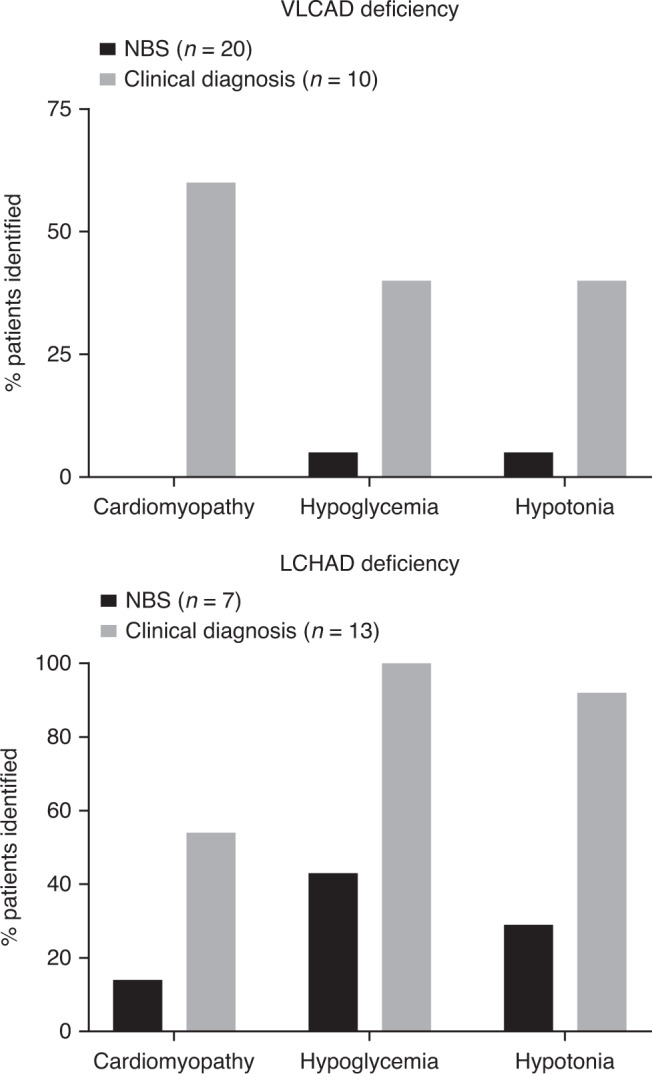
Rates of key clinical symptoms among patients identified by newborn screening (NBS) or clinical diagnosis. Patient numbers represent total number of patients identified by NBS or clinical diagnosis and rates of symptoms at initial disease presentation are based on numbers of patients identified in each group. Patients with multiple symptoms may be counted more than once. LCHAD long-chain 3-hydroxyacyl–coenzyme A dehydrogenase, VLCAD very long–chain acyl–coenzyme A dehydrogenase.

Widespread NBS availability allows patients with FAODs to be identified earlier than with symptomatic clinical diagnosis. Evidence from numerous retrospective analyses supports the notion that early NBS identification in the neonatal period has improved outcomes compared with patients diagnosed clinically after development of symptoms. One retrospective analysis examined 75 patients with LC-FAODs from centers in Germany, Switzerland, Austria, and the Netherlands.^46^ NBS-identified patients (*n* = 32) had a lower mortality rate (4/32 [12.5%]) than clinically diagnosed patients (8/29 [27.6%]). NBS-identified patients in this study also had lower rates of cardiomyopathy, hypoglycemia, and other complications at initial disease presentation than those identified by clinical diagnosis (Fig. 1).^46^ Multiple studies have recognized that NBS-identified patients with VLCAD deficiency can remain asymptomatic, likely due to preventive care, though the possibility of higher residual enzyme activity resulting in milder disease may also play a role. However, it is important to note that even patients with variants that are typically associated with mild outcomes such as VLCAD are at risk of sudden death and/or cardiac dysfunction with stress at any time or age.^47^

A retrospective analysis of 14 Austrian patients with LCHAD deficiency also found that complications (hepatic disease, cardiomyopathy, and retinopathy) were less frequent in patients identified by NBS (*n* = 9) than in clinically diagnosed patients (*n* = 5, including two patients with false-negative NBS).^37^ Although about one-third of patients identified as positive by NBS were symptomatic before NBS results were available, disease was not as serious as in a historical control cohort. In addition, growth and psychomotor development were normal in all except two extremely premature infants. Regarding management, all 14 patients received a fat-defined diet, and four also received supplementation with triheptanoin (marketed by Ultragenyx Pharmaceutical, Inc. as Dojolvi™) for varying durations, generally leading to clinical stabilization and reduction in creatinine kinase peaks.

A retrospective review at a single center in Seoul, Korea, compared clinical, biochemical, and molecular characteristics of patients with LC-FAODs identified by NBS with clinically diagnosed patients.^48^ Of 14 NBS-identified patients, 3 developed recurrent rhabdomyolysis or cardiomyopathy, and one died of cardiomyopathy. The remaining ten patients remained asymptomatic and showed normal neurodevelopment on follow-up. In contrast, of eight clinically diagnosed patients, six patients were diagnosed with LCHAD/TFP deficiencies after presenting with recurrent rhabdomyolysis or cardiomyopathy, one patient with VLCAD deficiency had cardiomyopathy, and one patient with CPT-1 deficiency had hepatic failure. Further, two patients with LCHAD/TFP deficiencies died due to cardiomyopathy in the neonatal period, and developmental disability occurred in one CPT-1–deficient patient. Prompt NBS (mean 3.2 ± 0.2 days after birth) helped to identify various FAODs, and thereby facilitated early intervention to improve clinical outcomes.^48^ Again the timeliness in screening, reporting of screening outcomes, and initiation of disease management play a considerable role in the prevention of potentially life-threatening metabolic decompensation events.^43–45^

With respect to long-term follow-up, a retrospective review in the Netherlands evaluated the effect of NBS on clinical outcomes 9 years after introduction in patients with VLCAD deficiency, given that many patients at diagnosis are asymptomatic.^49^ Of 57 patients identified with VLCAD deficiency (26 pre-NBS vs. 31 post-NBS), mortality was more frequent in patients identified pre-NBS (4 cases) compared with post-NBS (2 cases). Of 36 living patients seen during regular follow-up (17 pre-NBS vs. 19 post-NBS), hypoglycemia was detected in 10 of 17 (59%) pre-NBS patients compared with 1 of 19 (5%) post-NBS patients. Cardiomyopathy was present in 3 of 17 (18%) pre-NBS patients and no post-NBS patients. Myopathy was present in 15 of 17 (88%) pre-NBS patients and 2 of 19 (11%) post-NBS patients. Finally, neurological examination during regular clinical visits was abnormal in more pre-NBS patients (4 of 17 [24%]) than in post-NBS patients (1 of 19 [5%]).^49^ As in any study of NBS, the more favorable clinical outcomes among NBS-identified patients could reflect earlier management initiation or increased detection of patients with variants associated with mild disease course.

Currently, there are several LC-FAOD registries in place to monitor long-term outcomes associated with specific forms of disease or treatment options. These include the Inborn Errors of Metabolism Collaborative Stakeholder Network’s Information System (https://ibem-is.org/), the Newborn Screening Translational Research Network (https://www.nbstrn.org/), and the Unified European Registry for Inherited Metabolic Disorders of the European Reference Network for Hereditary Metabolic Disorders (https://www.u-imd-registry.org/). While many NBS programs collect follow-up data on identified patients, analyses of these data are limited. One such study reported that only approximately 55% of 426 patients diagnosed with LC-FAOD remained available for follow-up after 5 years.^50^ Another examined 52 patients diagnosed with LC-FAOD for an average of 2.4 years after diagnosis.^41^ In this study, 46 patients received molecular testing, identifying a total of 90 disease-causing alleles. Therefore, additional long-term follow-up studies are needed to fully understand the clinical spectrum of NBS-identified patients, establish genotype–phenotype correlations, and determine the effect of different management strategies.

A retrospective chart review of patients with confirmed metabolic disorders born before 2012 and followed at Boston Children’s Hospital compared clinical and neurological development in NBS-identified and clinically diagnosed patients.^51^ Of 101 patients with an FAOD identified through expanded NBS, 33 had MCAD deficiency, 15 had VLCAD deficiency, and 12 had carnitine uptake deficiency. Of 43 patients with an FAOD symptomatically identified before NBS, eight patients had MCAD deficiency, and three had VLCAD deficiency. Of 59 NBS-identified and clinically diagnosed cases involving either MCAD or VLCAD deficiency, 1 of 48 (2%) had serious disease compared with 1 of 11 (9%) clinically diagnosed cases. Mortality was substantially lower in NBS-identified patients (1 death) compared with clinically diagnosed patients (12 deaths). Unfortunately, the number of clinically diagnosed patients with FAODs who received developmental or neuropsychological testing was too low to allow comparisons with NBS-identified patients. However, among NBS-identified patients with MCAD and VLCAD deficiency, developmental quotient scores and full-score intelligence quotient scores were similar to that seen in the normal population, and >85% of patients performed in the average range (developmental quotient/intelligence quotient: ≥85).^51^

### Mortality

Although FAOD-related mortality data are available from numerous studies of symptomatically and NBS-identified patients, the small sample sizes limit conclusions (Table 3). Separate studies from China and Saudi Arabia exclusively examined NBS populations.^14,52^

**Table 3:** Mortality rate of FAODs overall and for specific disorders in selected studies/registries.

Country/registry	Year	VLCAD deficiency	LCHAD deficiency	CPT	CACT	TFP
**Asia/Oceania**						
China^14^	2009, 2016	0% (*n* = 0/3)		CPT-2: 100% (*n* = 2/2)		
India^69^	2017			CPT-1: 25% (*n* = 2/8)		
Japan^70^						43% (*n* = 6/14)
New Zealand^34^	2004–2009	0% (*n* = 0/1)			33% (*n* = 1/3)	
**Europe**						
Finland^71^			37.5% (*n* = 6/16)			
Finland^72^			94% (*n* = 15/16)			
France^53^	1977–1990, 1991–2000, 2001–2009	60% (*n* = 20/33)	63% (*n* = 26/41)	CPT-1: 25% (*n* = 1/4) CPT-2: 67% (*n* = 10/15)	92% (*n* = 12/13)	
France^73^	2014				65% (*n* = 18/28)	
Germany^17^	1999–2000	0% (*n* = 0/2)	LCHAD deficiency: 67% (*n* = 2/3)	CPT-2: 100% (*n* = 1/1)		
Germany^74^						16.7% (*n* = 1/6)
The Netherlands^30^	2007–2018	12.5% (*n* = 8/64)				
Poland^68^	1992–2009		36% (*n* = 21/59) NBS: 7%; clinical diagnosis: 45%			
Spain^73^	NR				50–65%	
**Middle East**						
Saudi Arabia^52^	2002–2016	62% (*n* = 23/37)				
Saudi Arabia^54^					29.4% (*n* = 15)	
**Multiple**						
Germany, Switzerland, Austria, and the Netherlands^46^	2009	6.6% (*n* = 2/30) NBS: 0%; pre-NBS: 20%	15% (*n* = 3/20) NBS: 0%; clinical diagnosis: 23%	40% (*n* = 5)		71.4% (*n* = 4/7)NBS: 67%;clinical diagnosis: 75%
United Kingdom and the Netherlands^75^	NR		38% (*n* = 19)			

*CACT* carnitine–acylcarnitine translocase, *CPT* carnitine palmitoyltransferase, *CPT-1* carnitine palmitoyltransferase-1, *CPT-2* carnitine palmitoyltransferase-2, *FAOD* fatty acid oxidation disorder, *LCHAD* long-chain 3-hydroxyacyl–coenzyme A dehydrogenase, *NBS* newborn screening, *NR* not reported, *TFP* trifunctional protein, *VLCAD* very long–chain acyl–coenzyme A dehydrogenase.

As with other outcomes, mortality rates varied among studies based on population and specific disorders, but were typically high. For example, overall mortality was 48% in a cohort of 187 symptomatic patients with MCAD (25%); LCHAD (22%); VLCAD (19%); or CPT-1, CACT, and CPT-2 (19%) deficiencies aged <6 years at diagnosis (between 1977 and 2009).^53^ Survival rate was significantly less favorable with younger age at disease onset, and with certain defects such as CACT deficiency (mortality: 92%) compared with other disorders such as MCAD deficiency (mortality: 20%). In support of this finding, a German cohort of 27 symptomatic patients (20 with MCAD deficiency) had a mortality rate of 19%.^17^ Lower mortality rates were generally observed in NBS-identified patient cohorts. This tendency was pronounced in LCHAD deficiency, which exhibited a mortality rate of 0–6.6% in NBS-identified patients compared with 23–93.8% in clinically diagnosed patients. In the same study, VLCAD-deficient NBS-identified patients had a markedly reduced mortality rate (0%) compared with symptomatically identified patients (20%). However, similar mortality rates were identified in screened and unscreened VLCAD-deficient patients in two other studies (0% for both in one, and 6% NBS and 15% symptomatically in the other).^30,34^ Mortality rates were similar in a small cohort of patients with CPT-2 deficiency identified by NBS and after development of symptoms. Interestingly, the only study including patients with VLCAD, LCHAD, TFP, and CPT-2 deficiencies identified both by NBS and clinically demonstrated higher mortality in NBS-identified patients (67% vs. 0%, respectively), but the sample size was limited to three and two patients in each cohort.^46^ No studies reporting mortality in NBS-identified patients with CACT deficiency were available for comparison.

## GENOTYPIC AND PHENOTYPIC SPECTRUM OF FAOD

Because a positive NBS for an FAOD does not always portend early-onset disease requiring immediate therapy, it is critical to distinguish through confirmatory testing patients likely to remain asymptomatic as newborns and young children from heterozygotes at no risk for disease. DNA sequencing is probably the most readily available follow-up test in most centers. Genotyping in the context of NBS sometimes provides valuable information on the nature and frequency of genetic variants involved with different FAODs and their association with specific phenotypes. Many pathogenic variants have been reported in LC-FAOD genes, including missense, frame-shift, and truncating variants.^52,54,55^ Additional variants are overrepresented in specific populations (Table 4).^54,56–58^ For example, in Saudi Arabia, VLCAD deficiency is commonly due to the presence of homozygous nonsense variants (c.65C>A, p.Ser22X) of exon 2 of the *ACADVL* gene and is associated with an early-onset phenotype.^52,54^ High allele frequency of specific variants has been found in other isolated communities, such as a predominant c.1436C>T (p.Pro479Leu) variant of the *CPT-1A* gene found in Nunavut Aboriginal populations in northern Canada and native populations in Alaska.^56,57,59^

**Table 4:** Common genetic variants in several FAODs and their reported allele frequency by country/region.

FAOD	Gene	Variant	Country/region	Allele frequency	Comments
CPT deficiency	*CPT-1*	c.1436C>T (p.Pro479Leu)	Northern Canada^56^	0–0.85	Highest frequencies noted in Nunavut Aboriginal populations
			Canada^57^	0.81	Nunavut region
			Alaska^59^	0.25–0.70	Highest frequencies noted among Alaskan Native populations
	*CPT-2*	c.338C>T (p.Ser113Leu)	South Italy^76^	0.53	Associated with homozygous or compound homozygous variants in most cases
			Italy^63^	0.68	Associated with adult-onset mild phenotype
			France^77^	0.68	Pathogenic gene variants present in either homozygous or compound heterozygous forms
			Germany^78^	0.80	
			United States^79^	0.95	Associated with homozygous (5/20) or heterozygous (14/20) forms
			European countries^61^	0.80	
LCHAD deficiency	*HADHA*	c.1528G>C (p.Glu510Gln) or (p.Glu474Gln)	Ukraine^80^	1.00	All homozygous (4 patients)
			Finland^71^	1.00	All homozygous (16 patients)
			Poland, Kashubian population^81^	0.99	Higher frequency of this allele may confirm founder effect in this population (heterozygosity rate: 1/57)
			The Netherlands^82^	0.87	Includes homozygous (25/34) and heterozygous (9/34) forms
			Poland^68^	0.91	Includes homozygous (45/59) and heterozygous (13/59) forms
VLCAD deficiency	*ACADVL*	c.65C>A (p.Ser22X)	Saudi Arabia^54^	1.00	All homozygous forms
			Saudi Arabia^52^	0.84	Other cases were of various nonsense variants
		c.848T>C (p.Val283Ala) or (p.Val243Ala)	United States^38^	0.58	
			Poland^68^	0.50	
			Germany^58^	0.40	Various other variants present, some heterozygous, with low residual enzyme activity
			Spain^83^	0.30	Some heterozygous forms associated with normal biochemical parameters
			Australia^39^	0.30	Various other homozygous and heterozygous forms noted
			Germany^55^	0.23	Homozygous for V243A; associated with low residual enzyme activity
		c.1349G>A (p.R450H); c.790A>G (p.K264E); (p.Cys607Ser) c.1144A>C (p.K382Q); c.1246G>A (p.Ala416Thr)	Japan^60,84^	0.08–0.18	

*CPT* carnitine palmitoyltransferase, *CPT-1* carnitine palmitoyltransferase-1, *CPT-2* carnitine palmitoyltransferase-2, *FAOD* fatty acid oxidation disorder, *LCHAD* long-chain 3-hydroxyacyl–coenzyme A dehydrogenase, *VLCAD* very long–chain acyl–coenzyme A dehydrogenase.

Genotype–phenotype correlations have been noted for certain variants of some fatty acid oxidation genes. However, to draw definitive conclusions is challenging given the limited availability of patient data from such rare diseases. Unsurprisingly, homozygous null variants typically lead to absent residual enzyme activity and serious phenotypes.^58,60,61^ The common c.848TNC (p.V283A) variant of *ACADVL* is a good predictor of late-onset muscular disease.^41^ A common variant of the *CPT2* gene (c.338C>T; p.S113L) leads to partial inactivation of the enzyme and onset of muscular symptoms in adolescence or young adulthood (usually not detected by NBS), while most other variants cause prominent neonatal or early infantile disease.^62^ Patients who are compound heterozygous for the late-onset allele and one early-onset allele typically develop symptoms later in later infancy or early childhood. A common 985A>G (K304E) *ACADM* variant accounts for ~70% of mutant alleles in NBS-identified patients.^1^ Of note, patients homozygous for this genotype have variable phenotypes, ranging from early neonatal death to asymptomatic adults. In contrast, a second recurrently identified variant (199C>T) appears to provide protection from clinical symptoms in combination with the common variant.

A few studies of FAODs reported that genotype–phenotype correlations were difficult to establish in the presence of compound heterozygosity, emphasizing the need for functional studies in NBS-identified patients.^55,58,63^ One such study reported that the frequency of the P479L variant of *CPT1* as high as 73–81% in Inuit patients. However, despite this high incidence rate, specific screening for this variant has not been implemented as it remains unknown whether it presents an increased risk of infant morbidity and mortality.^56^ Another study measured residual enzyme activity in samples from patients with VLCAD deficiency to correlate enzyme activity with clinical outcomes. While the authors suggest that an activity level of <10% presents a risk of symptomatic disease and patients with >20% activity may remain asymptomatic through life, they ultimately concede that a larger patient population is required to draw definitive correlations.^58^

Environmental factors further confound the correlations between genotype and phenotype. Countless environmental exposures and triggers have been shown to modify the fatty acid oxidation pathway, including the use of statins and nutritional supplements, aspirin, or viral infection.^64^ Depending on the pathogenic variant and genes involved, such factors can modify residual fatty acid oxidation and/or trigger symptoms. Distinct combinations of environmental exposures and pathogenic variants have the potential to interact differently, making it challenging to definitively identify genotype–phenotype correlations, especially given the small patient population with FAODs. Further, it is possible that these external factors can elicit potentially life-threatening symptom presentation in patients whose disease is the result of pathogenic variants typically associated with a mild presentation.

Overall, while second-tier molecular analysis of patients already identified by NBS is likely to ultimately prove useful in defining genotype–phenotype correlations, its current diagnostic utility may be hindered by limited accumulated data. Additionally, identification of variants of unknown significance require functional studies and long-term follow-up to form meaningful connections to patient outcomes. The predictive value of even common variants may be limited by confounders including environmental factors or synergistic variants that impact disease presentation. As molecular techniques become more widely utilized, large-scale analyses of long-term patient data will prove invaluable in defining the role of the genotype in patients with LC-FAOD.

## CONCLUSIONS

Epidemiologic studies of NBS populations indicate that the combined incidence of all FAODs ranges from 0.9 to 15.2 per 100,000. MCAD deficiency is the most common FAOD. VLCAD deficiency is the most prevalent LC-FAOD in most populations, followed by isolated LCHAD deficiency. The other LC-FAODs are rare in most populations. Comparative studies consistently reveal an increased incidence of all FAODs following NBS implementation. However, this finding raises the question of whether all NBS-identified patients will develop symptomatic disease. The need for follow-up testing to confirm and characterize the nature of specific FAODs has been increasingly recognized.^31^ In addition, quantification of false-negative and false-positive results in NBS and how to reduce their frequency remain a challenge. Genotyping for the detection of variants associated with FAODs, along with functional studies when novel variants are identified, are an essential part of NBS follow-up. Genotype–phenotype correlation is evident for some variants, but the presence of compound heterozygosity in most patients often makes outcome prediction difficult.

Studies included in this review generally demonstrated improved outcomes for NBS-identified patients compared with clinically diagnosed patients, including reduced rates of symptoms, neurodevelopmental impairment or delay, and death. Unfortunately, the patient numbers included in these studies are small and do not allow for review of management effects based on different genotypes or other factors to be considered. Future research focused on predicting disease presentation among identified patients who often remain asymptomatic, especially early in the disease course, but who are also potentially at risk of serious manifestations, including sudden death, is warranted.^42,48^ Reports of high mortality associated with FAODs are largely based on studies of symptomatic patients, and more recent evidence suggests that the introduction of NBS has had a beneficial effect.

## Supplementary information

Supplementary Table S1
